# A Multitiered Solution for Anomaly Detection in Edge Computing for Smart Meters

**DOI:** 10.3390/s20185159

**Published:** 2020-09-10

**Authors:** Darmawan Utomo, Pao-Ann Hsiung

**Affiliations:** 1 Computer Science and Information Engineering, National Chung Cheng University, No. 168, Sec. 1, University Rd., Minhsiung, Chiayi 62102, Taiwan; pahsiung@cs.ccu.edu.tw; 2 Faculty of Electronics and Computer Engineering, Satya Wacana Christian University, Jalan Diponegoro 52-60, Salatiga 50711, Indonesia

**Keywords:** anomaly detection, LSTM, SVR, k-NN, edge, fog, cloud, smart meter, imbalanced, K-means, HDBSCAN, AMI, DNN

## Abstract

In systems connected to smart grids, smart meters with fast and efficient responses are very helpful in detecting anomalies in realtime. However, sending data with a frequency of a minute or less is not normal with today’s technology because of the bottleneck of the communication network and storage media. Because mitigation cannot be done in realtime, we propose prediction techniques using Deep Neural Network (DNN), Support Vector Regression (SVR), and k-Nearest Neighbors (KNN). In addition to these techniques, the prediction timestep is chosen per day and wrapped in sliding windows, and clustering using Kmeans and intersection Kmeans and HDBSCAN is also evaluated. The predictive ability applied here is to predict whether anomalies in electricity usage will occur in the next few weeks. The aim is to give the user time to check their usage and from the utility side, whether it is necessary to prepare a sufficient supply. We also propose the latency reduction to counter higher latency as in the traditional centralized system by adding layer Edge Meter Data Management System (MDMS) and Cloud-MDMS as the inference and training model. Based on the experiments when running in the Raspberry Pi, the best solution is choosing DNN that has the shortest latency 1.25 ms, 159 kB persistent file size, and at 128 timesteps.

## 1. Introduction

**Figure 1:**
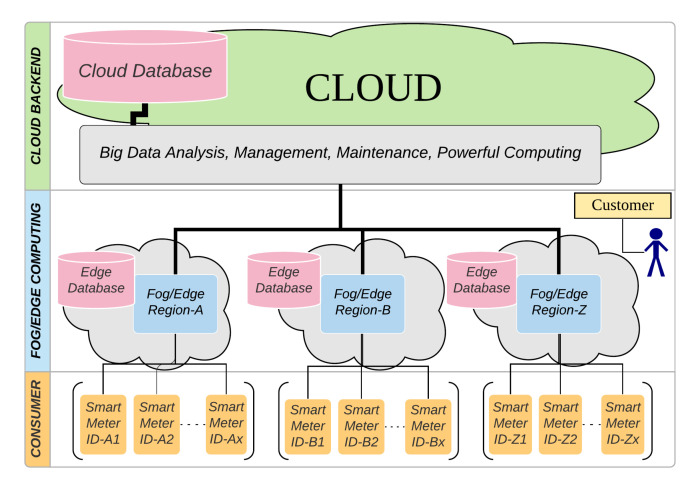
Cloud computing with edge/fog model in the smart grid.

Based on the predicted growth of the application of smart meters, it is estimated that, in 2024, there will be 1.2 billion smart meters in the world, with a cumulative expenditure of USD145.8 billion [1]. Utilities prefer Smart meters because of their ability to provide information more quickly and accurately optimize supply and demand. The smart meter in the smart grid system is also very suitable for integrating various energy sources. Traditionally, data from smart meters in the smart grid system are sent to cloud servers that have the ability to process large amounts of data and support big data analysis. However, this model has some disadvantages, such as difficulty in handling a large number of smart meters, and high latency, due to great distances, network loads, and additional layer to process the data. Therefore, data collected by smart meters are generally sent to Edge computing (EC) as an intermediate server between smart meters and cloud server. An EC device is a computer device that makes smart meters in the physical layer transparently connected to the computing world. Figure 1 shows an edge/fog computing general architecture that can be used in smart grid. EC is located near the applications, sensors, or data sources, but it may be far from the cloud. This is needed in order to reduce data exchange, shorten latency and response times, improve processing speed, and reduce pressure on the network as compared to connection through the cloud [2]. Data from smart meters are generally sent periodically at an interval between one to 60 min [3], for example, the dataset of London Low Carbon [4] has a frequency of 30 min. With a span of 30 min, EC can do data cleaning and data analytics that were previously sent to the cloud. In addition, EC can also activate security features through blockchain [5] and privacy [6], so that the quality of service is increasingly improved.

Smart meters according to its development are Automatic Meter Reading (AMR) and Advanced Metering Infrastructure (AMI). The capability of fully automated AMR is data transmission via fixed networks distribution to backend server without human intervention [7]. The information collected by AMR is usually for billing purposes only. AMI has the capabilities of AMR, coupled with two way communication between users and utilities, and also automated software that supports Meter Data Management System (MDMS) operations. Traditional AMI uses a centralized MDMS architecture [8] which makes it difficult to build it in large scale. With the EC and a more varied communication service provides many advantages such as online information to customers per half hour [9] and anomaly of electricity usage. Information that is actively provided to customers is intended to reduce unnecessary electricity usage, reduce electricity demand, reduce carbon footprint, etc. In this paper, AMI with EC architecture is discussed.

Different kind of analyzes such as Load Management, Load Analysis, Connection Verification, and Load Forecasting (LF) can be done at the EC level. This LF is divided up at the level of distribution of large areas such as cities, feeders, and final customers. End customers can be further divided into Industries, Businesses, and Residential. Higher loads will occur in some seasons, for example, in summer, due to air conditioning. Inadequate anticipation of anomalies can lead to cascading events that lead to blackouts.

The majority of blackouts around the world are caused by weather, faulty equipment, vehicle, animals, and over demand [10]. The cause of over demand can occur as a result of power imbalance between demand and supply, which will directly affect frequency stability, voltage reactive power, and synchronization of the entire power system. When an anomalous situation occurs, overload, tripping, and generator outage that are not addressed within a certain time will allow cascading events that can trigger blackouts. This occurred in Victoria, Australia during their summer, on 18 January 2018, for example, when an anomaly in electricity demand surges resulted in the failure of one of the coal plants to generate electricity [11].

In systems connected to smart grids, smart meters with fast and efficient responses are very helpful in detecting anomalies in real time. Smart meters used as smart sensors can be used to measure system parameters, such as power usage, transmission line temperature, and power outages and communicate between the generator and the user to ensure full network system capability. An anomaly detection algorithm [12] was developed to test the effectiveness of the frequency of the smart meter sending data on a smart grid that is simulated in MATLAB/Simulink. The test is carried out with a frequency of data transmission of 1, 15, and 30 min. The conclusion is that the more often a smart meter sends data, the more effective fault over voltage is detected. With faster data transmission from smart meters to EC, EC is able to process and analyze data faster.

However, sending data with a frequency of minutes is not normal with today’s technology because of the bottleneck of the communication network and storage media. In general, a residential utility network uses a time step of 15, 30, and 60 min and a commercial/business network every 15 min [13]. If mitigation cannot be done in real time, then prediction techniques are needed such as using the Kalman Filter [7] or by using Machine Learning (ML). In addition to these techniques are to extend the prediction time step to a longer one i.e., per day. The predictive ability of ML is applied here to predict whether anomalies in electricity usage will occur in the next few weeks. The aim is to give the user time to check their usage and from the utility side it is necessary to prepare a sufficient supply.

The four main problems associated with smart meter anomaly data in the EC must be resolved before predicting the consumption of electricity anomalies. First, the number of smart meters is too many for individual analysis to categorize, predict, and modify consumer’s energy consumption, and it is infeasible to generate a model for every household. Therefore, they must be processed in clusters, as discussed in [14]. This paper explains how to reduce the number of clusters by using dynamic time wrapping (DTW). Second, the number of normal cases far exceeds the number of anomalous results so techniques are needed to solve the imbalance. The proposed solutions include data preprocessing strategies (i.e., Random Under/Over-sampling, clustered based, synthesizing new data), special-purpose learning methods (Adaboot, P-SVM, KNN), and hybrid methods (i.e., SMOTE-SVM) [15]. Third is that the prediction performance of short datasets is lower due to lack of training data. For countries that are starting to install smart meters, it will be difficult to get a dataset that matches their environmental conditions. Multi-annual data will greatly help ML generalize short and long term memory, so that it can produce more precise predictions. The fourth problem is the performance, especially related to the speed of processing and the size of persistent file model. When doing the inferencing in the EC, the lower inference time will reduce the latency and the smaller persistent file size model will reduce the transmitting and updating model from cloud to EC.

**Figure 2:**
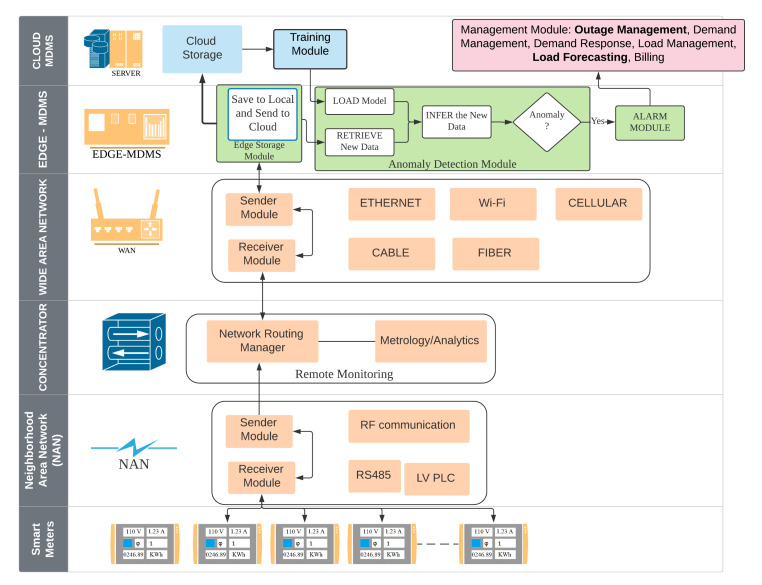
Anomaly Detection System Design.

Therefore, we propose a multitiered solution (MS) to group the relevant members, label expansion to increase minority samples without having to add new synthetic data samples, serializing data from the same cluster to overcome the lack of training datasets that only consist of one cycle, and determination the right type of learning machine by comparing the performance of neural networks especially long-short term memory (LSTM) that support time series data regression, Support Vector Regression (SVR), and k-Nearest Neighbors (KNN) in detecting anomalies. All the stages of this process are carried out on the Edge and Cloud Layer, as illustrated in Figure 2. The result of this multitiered solution is the inference model. Each inference model generated by the Cloud and sent to Edge is the result of training in the Cloud from the data collected from each Edge. If the clustering mode used is the intersection Kmeans and Hierarchical Density-Based Spatial Clustering of Applications with Noise (HDBSCAN), then Cloud performs clustering based on Kmeans and HDBSCAN. The resulting new clusters are then trained to produce model weights which are stored in the form of persistence files. This persistence file is sent to each Edge that contributes to cluster formation.

The rest of this paper is organized, as follows: Section 2 explains the related work various aspects of the problem and how others have attempted to solve each. Section 3 describes the design of an Anomaly Detection Architecture, the processing of the dataset, and the novel techniques developed. Section 4 explains the experimental setup and demonstrates situations in which the current proposed model performs better than existing structures. Section 5 highlights the most important contribution.

## 2. Related Works

This section will explain the handling of the four previous problems that are the focus of this work, namely, model reduction, techniques for expanding labels, expanding data points, and comparing anomaly detection in machine learning.

### 2.1. Model Reduction

Data from a smart meter are time series data. Ideally, for each ID meter a model is specially designed for that ID. However, this will make the number of models handled too large to handle the number of smart meters. From this time series data, the behavior of similar customers can be grouped based on electricity consumption. Thus, clustering methods are needed as long as the assumptions are valid. Many clustering models have been used, with two of the most common being Centroid models and Density models. Centroid models may be based on fuzzy c-means [16] or Kmeans [17], whereas density models may be a combination of principle component analysis (PCA) and density-based spatial clustering of applications with noise (DBSCAN) [18].

Kmeans clustering is often used because the time and space complexity are O(n k l) and O(k + n), respectively, where n is the amount of data, k the number of clusters, and l is the iteration that converts the algorithm. The weakness of Kmeans is that it has to determine the number of clusters from the beginning and the point of initialization can cause the solution to go into local minima [19]. On the other hand, DBSCAN focuses on the same density cluster [20] and it has an O(nlog n) performance if the R*-tree acceleration algorithm is applied. Moreover, the memory capacity without distance matrix is only O(n). The advantage of this algorithm is that it can separate noise, so that more similar cluster members can be obtained.

Anomaly detection processing with machine learning is very dependent on iterations. Processing too much data at once will be very burdensome for computing with high order complexity. Therefore, clustering is very important to limit the amount of the relevan data before being fed to machine learning. The focus of Kmeans and DBSCAN is different in terms of the partition area and density level. Therefore, we propose a combination of these two clustering to get more similar members, using a intersection technique which we call Kmeans*HDBSCAN.

### 2.2. Imbalanced Dataset

In supervised learning, the ideal condition for training is when the number of labels from each class is the same so the chance of each class in the learning process is the same. However, in the case of anomalies, the number of anomaly labels is always a minority when compared to normal or majority conditions. Two commonly used techniques to overcome this are oversampling minority and undersampling majority. Given the number of samples in imbalanced cases, oversampling is generally used, such as a synthetic minority over-sampling technique (SMOTE) algorithm [21] and its derivatives, such as adaptive sampling (ADASYN) [22]. However, Batista et al. [15] concluded that, for three minority/majority samples 1/99, 5/95, and 10/90 in five strategies, including SMOTE and ADASYN, did not yield good results.

The idea of SMOTE and other oversampling techniques is based on creating a cloud of samples between two real data [23]. If one real data is apparently on the wrong side, many high-bias samples tend to be generated. Thus, preprocessing must be handled before doing oversampling using this method. However, in the proposed method, we increase labels instead of feature data. The data contained in the smart meter are time series data. Anomaly labeling based on energy consumption is also based on time series. To predict anomalous events, pre-labeling is applied along the prediction range specified. For example, desired outcome may be the ability to predict seven days before. From the dataset it is known that there are anomalies on day 100. Thus the anomaly labels are expanded from day 94 to 100. In this way, the number of anomaly labels will increase according to predetermined prediction ranges.

### 2.3. Data Samples

A survey paper [24] states that a load profile that can predict weekly, daily, and hourly load consumption, which includes the Very Short-Term and Short-term Load Forecasting groups, is highly needed. To predict future events, previous observation points are required. For example, to predict a load with a time-of-day model generally takes from 24 h to 7 × 24 h, depending on forecasting expectations. This means that 24 to 168 data points are demanded per period. In daily cases, data points from the previous 24 days to 168 days are used.

However, the load profile from smart meter technology has not been implemented in all countries, so the data is still dependent on open datasets. The time span of a smart meter open dataset is often limited to one year or 365 days i.e., in [25]. This dataset consists of 200 smart meter IDs in one year. This is not enough to predict a whole year on a daily basis, since part of the dataset is used as observation points. To overcome this, we propose a clustering technique. All the smart meters incorporated in the cluster are assumed to originate from the same smart meter. In this way, the amount of data from the smart meter will increase significantly.

### 2.4. Comparison of Anomaly Detection in Smart Grid

Various techniques for detecting anomalies in smart grids have been studied and discussed, such as KNN [26], Support Vector Machine (SVM) [27], and LSTM [28]. These algorithms also discussed in the area of Internet of Things at the network edge [29]. For EC use, the latency time parameter becomes an important measure. Therefore, a comparison of these three algorithms will be observed in the following experiments. In terms of determining the model, training objectives, factor amount of data to the size of the model weight, training time, inference time shown in Table 1.

For applications with EC, inference time is very important, because it determines latency time. For this reason, we need a comparison of the latency times of the three models obtained from the testing process performed on the EC device.

## 3. Anomaly Detection System

**Figure 3:**
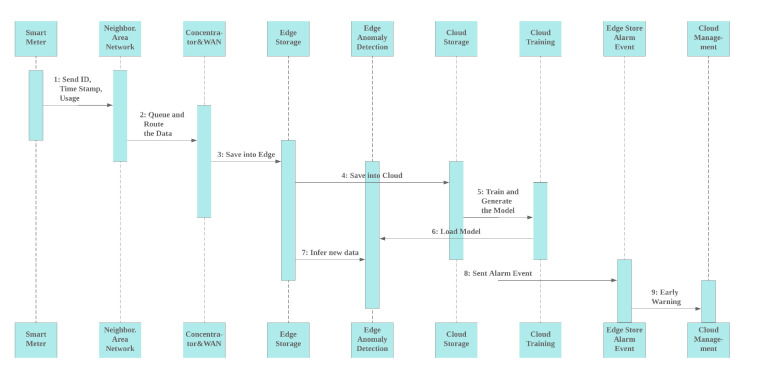
Sequence Diagram of Anomaly Detection System.

Figure 2 shows an anomaly detection system architecture. System is divided into six levels including Smart meters, Neighborhood Area Network (NAN), Concentrator, Wide Area Network [8], Edge-Meter Data Management System (Edge-MDMS), and Cloud-MDMS. At the lowest level, the Smart meters in one region periodically send data to the Concentrator via NAN. Within the Concentrator, data is redirected to the corresponding EC that will process this data through WAN. The contribution of this paper is the anomaly detector of electrical energy consumption by enriching the Edge and Cloud levels, namely EC-MDMS and Cloud-MDMS. EC-MDMS has five modules that are used to store data locally and also send them to the Cloud-MDMS, load models sent by the Cloud-MDMS, retrieve the inference data from local storage, process inferences, and send alarm signals when anomalies occur. This alarm signal is also sent to the Cloud as an early warning of anomalies. Periodically, the data contained in the Cloud-MDMS level storage are retrained to obtain a model that is in accordance with the latest conditions. New models that are generated periodically will be sent back to the Edge-MDMS level. Figure 3 shows a sequence diagram of how modules interact with each other to form an ecosystem of anomaly detection system.

Smart meter is a critical part and should be maintained carefully. It contains not only precision instrumentations, but also obligate sending the data and receiving the control on behalf of MDMS securely and privately. Recently, cyber-security has become prominent so that every smart meter must be equipped with high enough security facilities [35]. Apart from that, there are other matters that need to be addressed, such as tamper/theft detection, dynamic pricing, power outage detection, etc. With so many functions that need to be handled but with limited computational capabilities for a range of applications up to a dozen years, the possible pre-processing should be as simple and secure as possible. In general, to process large data to get features and data from data, an upgradable and scalable machine is needed, which, in this case, is simpler if processed in the Edge than replacing all installed smart meters.

**Figure 4:**
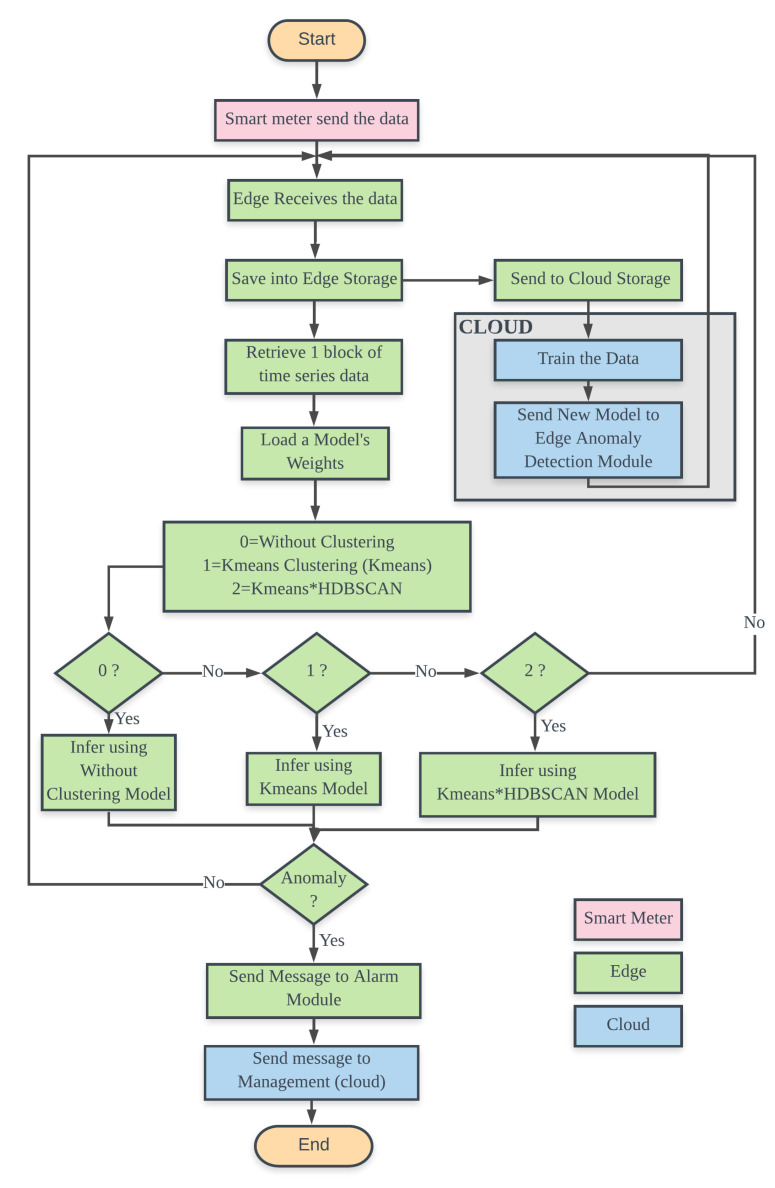
Anomaly Detection System Flowchart.

Figure 4 displays the flowchart of the Anomaly Detection System. The pink, green, and blue colors represent the location of the functions in the smart meter device, edge, and cloud. Inference on three different models, Without-Clustering, Kmeans, and Kmeans*HDBSCAN, can be performed by setting the corresponding Mode value. If the clustering technique is used, the identification number of the smart meter determines the location of the cluster model.

### 3.1. Dataset

The dataset from [25] was obtained from 200 households in the Midwest region of the United States. This data set contains clean data with a time stamp every 30 min from 1 January 2012 to 31 December 2012. In this experiment, the time stamp is accumulated to a day to accommodate daily consumption data and then normalized. An anomaly label is statistically determined by calculating the mean plus three times standard deviations [36,37].

**Figure 5:**
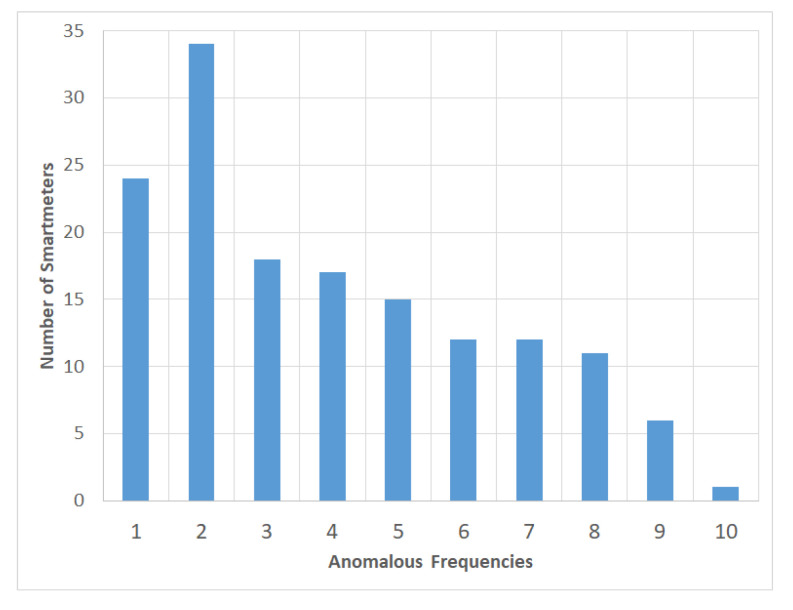
Observation of the frequency anomalous events from the total 150 smart meters.

Figure 5 shows the histogram of the anomalous frequency. Labels with anomalies were obtained as many as 597 scattered among 150 smart meters, whereas the other 50 smart meters do not have an anomaly label. Among 150 smart meters, 24, 34, and one smart meter each has one, two, and ten anomalous events. The 597 anomaly labels are only around 1.1% and is included in the highly imbalanced dataset domain, since it is located near with 1%. By convention, the ratio of imbalance class lies between from 1:4 up to 1:100 [38].

**Figure 6:**
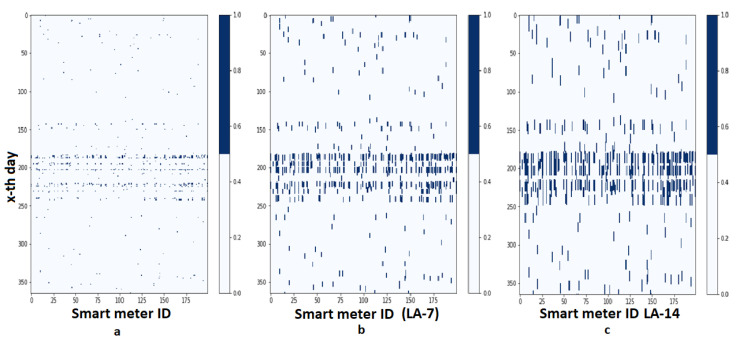
Anomaly Map Number of Smart meters and Day Number (**a**) Original statistical model, (**b**) With Look-ahead seven days (LA-7), (**c**) With Look-ahead 14 days (LA-14).

Figure 6 shows consecutive label data that is formed based on statistical calculations, LA-7, and LA-14. These labels have only two values, 0 and 1, representing normal and abnormal conditions, respectively. It is clear that expanding the label with LA will reduce the imbalance ratio label of anomaly and normality. The ordinate and axis represent the day number, starting from 1 to 365 and smart meter number. The months between June to August, which are equivalent to index 180 to 240, are summer season in the northern hemisphere. The electricity consumption normally increases in this period of time, with most used for cooling.

**Figure 7:**
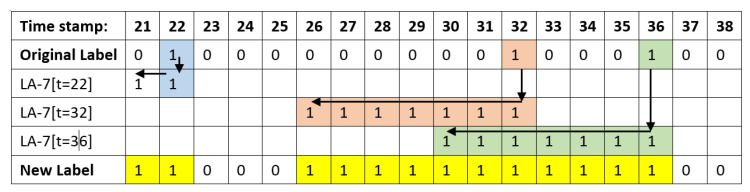
New Labels generation with LA-7.

Figure 7 shows how to expand the anomaly labels. The anomaly labels are originally located in sparse areas. The labels will be extended to seven days in order to compensate for minority samples before the events happened (LA-7). The original anomaly labels reside at t = 22, 32, 36. For example, the anomaly label at t = 32 (light orange) is expanded from t = 26 to t = 32. After expansion the anomalous label, the third to fifth rows show how the anomaly labels are OR-ed to yield the New Label, as seen in the last row.

**Figure 8:**
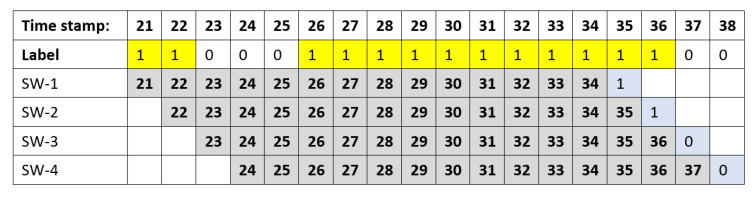
Sliding Windows Data generation with timesteps = 14.

In Figure 8, sliding window data are generated by combining the sequences of data values, in this case each timesteps is 14 days, with a label, (light blue) from next timestamp. Rows SW-1 up to SW-4 show how four sliding windows data are generated. LA-X is varied to 7, 14, 21, 28, and 35, the number of anomalous labels will increase by 6.2%, 10.9%, 14.9%, 18.2%, and 21.3%, respectively. This percentage represents random sampling probability without prediction techniques.

Three types of datasets are prepared for Without-Clustering, Kmeans, and Kmeans*HDBSCAN. These datasets will be trained and tested within a combination of Convolution1D and LSTM [39] provided in main Deep Neural Networks (DNN) frameworks, KNN [15,29], and SVR [31]. The performance goal is indicated by the higher ROC-AUC, PR-AUC, and the processing time for each prediction step. The preparation of three types of datasets is arranged before serialization. After these three types of datasets are formed, serialization will be carried out, which will be explained in the Section 3.3.

#### 3.1.1. Without Clustering

This time series dataset needs to be separated for training and testing, which are determined at 80% and 20%, respectively. The number of rows of 365 samples is too small for the training process, even though a sliding windows technique was used. To overcome this problem, it is assumed that all smart meters from the resident households and neighboring smart meters are essentially homogeneous. Each dataset from the smart meter is stacked with the previous smart meter data. With this assumption, the entire serialization of the dataset is 73,000 × 1 sample. These data are sufficient to be separated into training and testing datasets. Henceforth, it will be referred to as the Without Clustering type.

#### 3.1.2. Kmeans Clustering

Assumptions of homogeneity are not necessarily true, but can still be corrected by grouping the dataset according to the closest cluster. One of the most widely used clustering methods is Kmeans. A total of 200 smart meters were clustered with Kmeans for two to 10 clusters and tested with Silhouette Coefficient (SC) [40]. SC gives a weight close to one if the resulting cluster has members that are close to the center of the cluster and negative one if the members of the cluster are closer to other clusters. From this test, only two clusters are recommended with SC values of 0.301. The range of silhouette values ranges from –1 to +1, where the value +1 indicates that the object matches its own cluster and does not match the neighboring group. The SC value of 0.301 indicates that there are many points that match the cluster, but there are also some that do not match. Algorithm 1 explains how to determine SC obtained after separating data from two to NMAX+2 clusters. After the cluster is determined, the data need to be splitted into appropriate clusters. This is done in Algorithm 2. This algorithm combines the similar smart meter IDs in one sub-dataset.
**Algorithm 1 **Best_K_Cluster: Finding the best k of Kmeans’s clusters.
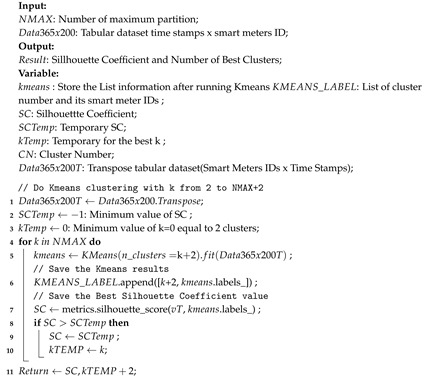


**Algorithm 2 **Kmeans: Choosing members, data, and labels of Kmeans Clustering.

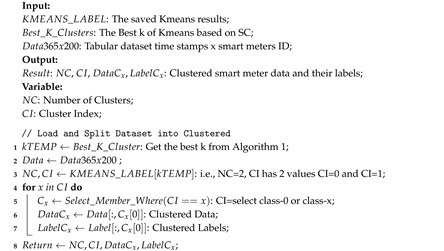



#### 3.1.3. Kmeans*HDBSCAN Clustering

We combine the Kmeans and HDBSCAN. Kmeans is basically a partition that assumes data are collected in spherical form, equal size and dense, densest in the center of the cluster, and does not include noise or outliers. However, this real dataset has noise that should be removed before the learning process. Therefore, we apply HDBSCAN, which has the capability to determine noise and cluster data [41].

**Figure 9:**
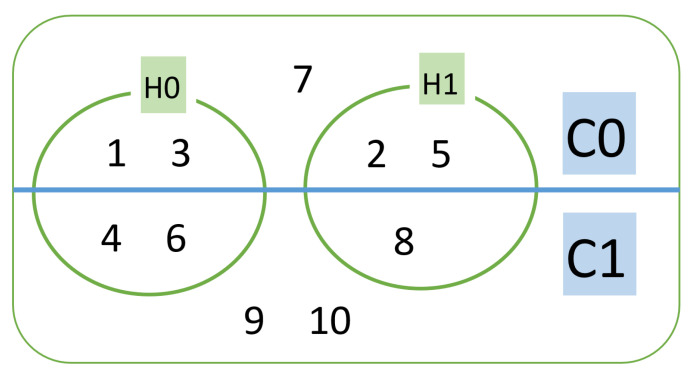
Illustration of intersection of Kmeans and Hierarchical Density-Based Spatial Clustering of Applications with Noise (HDBSCAN) Clustering.

In Algorithm 3, the Kmeans*HDBSCAN process is carried out. In this process, all of the clusters from HDBSCAN are intersected with all clusters from Kmeans. Because it is mutually exclusive, it is not possible to have one ID occupying two sub clusters of Kmeans*HDBSCAN. Figure 9 illustrates Kmeans that has two clusters C0 and C1, while HDBSCAN also produces two clusters H0 and H1, and also cluster noise. In this case C0H0 has ID-1 and ID-3 smart meters. Whereas the noise cluster members are ID-7, ID-9, and ID-10.
**Algorithm 3** Kmeans ∩ HDBSCAN.
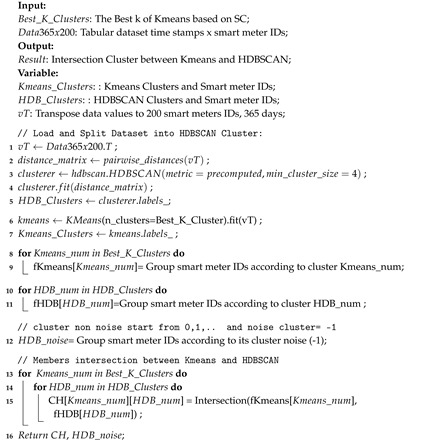


### 3.2. Platform

**Figure 10:**
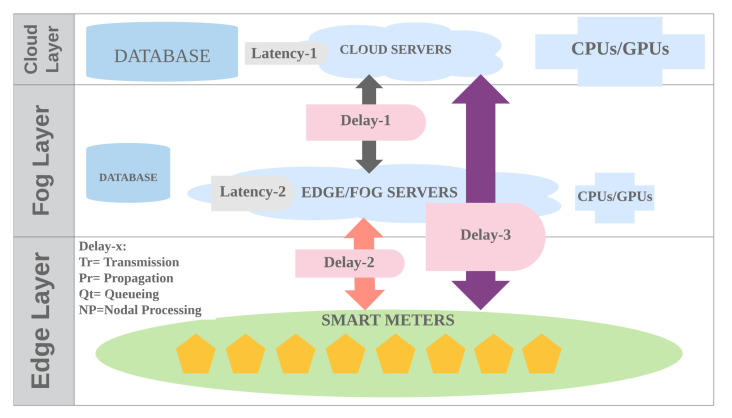
Delay and Latency in fog/edge and cloud server architecture.

Transmission delay, latency in data processing, and deadlines are the main factors in the design of systems with smart meters. Transmission delay that is too large requires an fast edge/fog server and cloud to reduce latency, so that targets from deadlines can be met. A paper discusses the effect of the average delay ratio between fog and cloud with the parameters of the number of server fog ratios four times the cloud, the fog processing power ratio of 10% of the cloud [42]. The variable is the delay fog to cloud ratio that is changed from 1% to 85%. From Figure 10, this delay ratio is Delay-2 to Delay-3. Delay-x is a combination of various kinds of delay, such as transmission delay, propagation, queueing, and router/nodal processing. A 1% delay ratio means that the smart meter device is very close to the edge/fog server and the location of the cloud is relatively very far. From this experiment 100 s total latency was found that the cut-off point when the average delay ratio was 85% and at the data request 5000 packets. The average number of datasize requests greater than the intersection indicates that the latency of the edge/fog is closer to the cloud and very far from the smart meter device. In this experiment, the Latency-1 of the cloud was determined at 10 ms. Therefore, in this study measurement of Latency-1 and Latency-2 as parameters when deployment of this system to the actual application is needed and it will be discussed in Section 4.2.5.

The latency measurement algorithm is done during the testing process. The average time difference before and after inference is defined as latency. There are two latencies that are sought, in this case Latency-1 and Latency-2, which are located on the PC server and edge. The Algorithm 4, shows how the measurement process is applied. The PC server and edge represented as Raspberry Pi both run in stand alone mode to reduce disturbing from another process.
**Algorithm 4** Latency (ms) measurement in edge and cloud.
 **Input**: 
   TestingDataset;
   model ;
   datetime;
   Number_of_Testing_Samples;
** Output**: 
   Result: LPS= Latency per sample (ms);
** Variable**: 
* T*_old: Starting time;
* T*_now: New time;
   Total_Latency: Total Elapsed Time;
 // Close other programs
**1**
*T*_old ← datetime.now();
 // DNN/SVR/KNN model
**2** Do model.predict(TestingDataset) ;
**3**
*T*_now ← datetime.now() ;
**4**
Total_Latency ← *T*_now – *T*_old;
**5**
LPS ← (1000*Total_Latency/Number_of_Testing_Samples);
**6**
Return
LPS;


### 3.3. Methods

**Figure 11:**
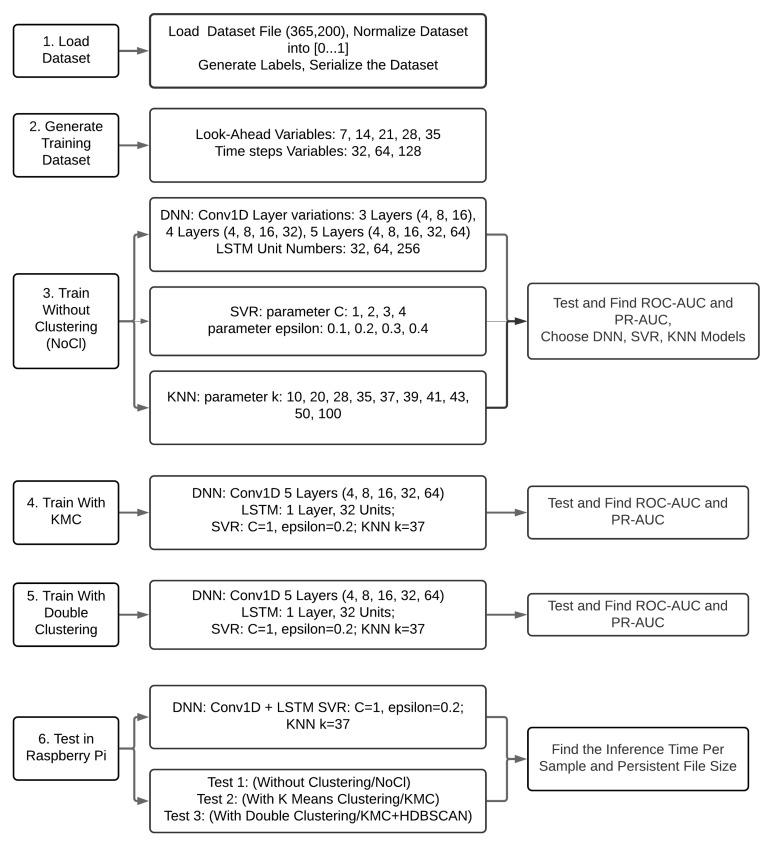
Experimental Setup Design.

The experimental steps are shown in the Figure 11 and explained, as follows. The first step is to load the original dataset. The original dataset is a collection clean data from 200 smart meter IDs, with 30 min time stamps from one year. This dataset is rearranged by accumulating it from 30 min to one day in order to conform with the day as the domain prediction. This dataset is then normalized to the maximum value of each smart meter. The size of this dataset is shaped into 365 days within 200 IDs (365, 200). The second process is to create labels based on the statistical definition of mean+3.standard_deviations. The size and shape of these labels are the same as the dataset. This step is continued with the serialization process, which is changing the shape from (365, 200) to (73,000, 1) by stacking each data and label of each smart meter.

This first step has two others variations, namely serialization with clustering, which is clustering all of the data into two clusters and serialization with Kmeans*HDBSCAN. The difference with and without clustering is the selectivity of the smart meter data to be trained and tested becomes narrower. Clustering all if the data into two clusters split the 200 smart meters of data into two clusters. The clustering method applied is Kmeans with a value of k = 2. The resulting clusters have 123 and 77 smart meters.

The second step is to prepare the dataset, so that it can be processed by machine learning, which in this case is Conv1D-LSTM, KNN, and SVR. The format requested by LSTM is (samples, time, feature). The feature used here is only one, electricity usage. Time represents the number of time stamps or timesteps used to predict output. Samples states the number of data samples that are already in time series format. Time is composed of sliding windows features. In this experiment the feature or data width of the sliding windows used is 32, 64, and 128. The width of the sliding window label is determined at 7, 14, 21, 28, and 35 where represent one to five weeks’ prediction. The purpose of this sliding window is to label the anomaly several timesteps before the predicted anomaly. The second goal is to increase the label anomaly, so that it reduces the imbalance rate. The algorithm used to generate labels and sliding windows is described in Algorithm 5. This algorithm has been used to read sequence data and convert it to standard LSTM data and an output vector [43]. The difference with this algorithm lies in the line number 9, where the output will be labeled 1 as long as there are anomalies in the range.
**Algorithm 5** To_Serialization: Generating Labels and Sliding Windows.
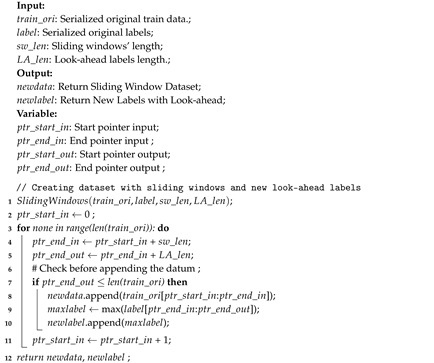


The third step is trying to find the good hyperparameters in DNN. The simplified DNN model to find the hyperparameters is presented in the Algorithm 6. The first trial is checking to find the epoch number for training. The experiment start to find a relative good performance in a relative small training time, since a small epoch would save the training time. Here, some initial observations to get the epoch value from the training are determined from large to small numbers from 800, 400, 200, and 100 to get a picture to a more detailed picture. Epoch 400 shows the results of a stable fitting, not much different from epoch 800, and it does not too fluctuate as in Epoch 100. Therefore, epoch 400 will be used in all of the experiments related with DNN.
**Algorithm 6** Finding the best parameter for DNN.
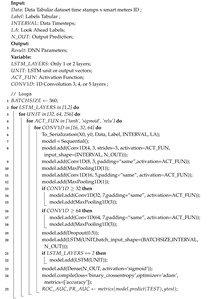


Other parameters tested are the number unit/tensor of LSTM (32, 64, 256). This unit represented the dimensional output. The next parameter is Convolution stack (3,4,5), where, from this experiment, the good features based on the Maxpooling can be found. The best candidate model is chosen based on the best performance. Subsequently, this candidate model was tested again with stratified cross validation k = 5. Stratified cross validation k = 5 will divide the anomaly label proportionally 80% for training and 20% for testing. In experiments with cross validation k = 5, training was conducted five times. The results from the ROC AUC metric are averaged to represent this experiment. Furthermore, the best model is tested again for variations in the activation function at the last layer (Sigmoid, ReLU). The expected performance according to EC is an accurate prediction seen from the highest ROC value and the shortest prediction time.

The two other sub-steps are applying KNN and SVR using the same dataset to find the ROC-AUC of each algorithm. The KNN experiment was done by choosing the best k by manual selection after the ROC and PR were calculated and summed to yield only one value. Here the k was found with sequences 20, 50, 35, 28, 43, 39, 37, 41, and 100. The SVR’s main parameters that will be treated as variables are C and ϵ. Parameter C is changed from 1 to 4 and ϵ from 0.1 to 0.4. After the training, all of the model weights are saved into files to select the best results as the model comparators to the DNN.

The fourth and fifth steps involve repeating steps three but serializing with Kmeans and serializing with Kmeans*HDBSCAN. The parameters chosen in the fourth step will be used again here.

The last step is loading the model into a Raspberry Pi to find the processing time. Three persistent models from DNN, KNN, and SVR are selected and transferred to Raspberry Pi. Each model was run and the processing time during inferencing observed. These results will show the performance of each algorithm in the Edge.

## 4. Evaluation

### 4.1. Experimental Environment

The experimental environments are shown in Table 2. This table shows the hardware and software that are used in this experiments. A standalone PC-server is connected to the Raspberry Pi in a dedicated LAN. Raspberry Pi is chosen as an Edge, mainly because of library support and compatibility with ML operations and the Tensorflow framework.

### 4.2. Results

The experimental results are explained in three subsections, namely Without Clustering, Kmeans, and Kmeans*HDBSCAN, as follows.

#### 4.2.1. Without Clustering Experiment

At the beginning, the input data are going to be processed in the Convolution and Maxpooling layers. Both of the layers are utilized to find the maximum keypoints among the data points. Convolution will increase the number of parameters and Maxpooling will decrease the number of parameters. Maxpooling 1 × 3 will reduce the number of calculations to only 1/3 of the output parameters. This parameters can be calculated manually or by running a Keras function model.summary().

The output parameters then feed into LSTM. One of the LSTM hyperparameter is Unit. The LSTM Unit number determines the number of output dimension or Tensor, the size is linear inside LSTM layer. For the two LSTM layers, the output parameters from layer one are multiplied by the unit numbers in layer two. This causes the number of variable tensors to be processed to be high if the parameters on the previous layer are large.

**Figure 12:**
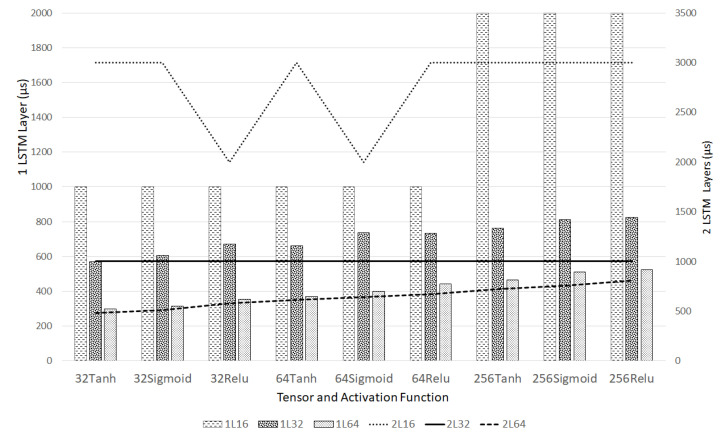
Training time per step for 1 and 2 layer long-short term memory (LSTM) with 32 upto 256 LSTM unit, with Convolution channel 16, 32, and 64, and activation function Tanh, Sigmoid, and ReLU.

From Figure 12, the horizontal axis represents the experiments by changing the unit numbers and activation functions. Each axis point has six experiments, three each for 1 and 2 LSTM layers, represented by bar and line graphs, respectively. For example, 32Tanh shows three experiments with single-layer LSTM with convolution up to layers 16, 32, 64 symbolized by the dashed-1L16, dotted-1L32, and light-dotted-1L64 bars, respectively. When 1L64 and 2L64 are compared, they look similar but the ordinate time scale of 2L is 1.6 times that of 1L. This experiment confirms that 2L will require more CPU power that may not be suitable for running in EC. Regarding a convolution layer, 1L64 is faster than 1L32 and 1L16. This experiment also shows that the longer layer will fill many points with zeros since the maximum values are more preferred. Thus making the calculation with zero is faster. Hence, the 1L64 is faster than 1L32 or 1L16. The next step is to try to find the best ROC-AUC and PR-AUC result.

Table 3 shows the ROC-AUC, PR-AUC, and the sum of ROC-AUC and PR-AUC results. The highest ROC-AUC experimental result is yielded by N64-CV64 with the Sigmoid activation function that obtains score 0.885. N64-CV64 is the abbreviation of LSTM with 64 Unit or tensor and five Convolution layers (4, 8, 16, 32, 64). The average training time per step of model N64-CV64 for Tanh, Sigmoid, and ReLU are 371 μs, 400 μs, and 442 μs, respectively.

The best PR can be obtained using N64-Cv64-ReLU which has AUC PR of 0.595. The best sum of ROC-AUC and PR-AUC is found using N32-Cv64-Sigmoid with 1.465, which is the best result in this table. If inference speed is important in the Edge, model N32-Cv64-Tanh with a score of 0.852 is the best choice. For this reason, N32-Cv64-Tanh is chosen for subsequent experiments.

The number of imbalanced labels are expanded by adding labels before the original anomaly label based on the assumption that the system must be able to warn of the anomaly in previous timestamps. In this experiment, changing the label to an anomaly was done in 7, 14, 21, 28, and 35 days before the anomaly occurred. This change will increase the number of labels without changing the input data.

Table 4 shows the test results of one layer LSTM with timesteps 32, 64, and 128. Normally, increasing timesteps would increase the performance, because the network is given more data to do estimation. Table 4 confirms the hypothesis for ROC-AUC and PR-AUC. The smaller time step is faster, because less memories are included in the calculation. However, in the next experiment, we want to do performance comparison with the other model. That is why in this experiment time step 128 is preferred. Intuitively, the longer LA will increase the anomaly detection probability. It implies increasing the PR-AUC performance since the increasing of True Positives, as shown in Table 4. The difference between D200-064 and D200-128 is not very significant, but it only requires half of the sample size, so that the amount of time used to predict is also shorter. However, if the memory and processing time are still sufficient to fulfill the specification, the D200-128 that performs slightly better should be chosen.

The above experimental results were compared with SVR and KNN. SVR has two hyper-parameters C and ϵ. A limited grid search is chosen in order to find the parameters that infer the good results. Table 5 is obtained from the results of experiments with parameters C = 1 to 4 and ϵ = 0.1 to 0.4. The dataset is the same dataset as experiment DNN without clustering. Most SVR result performances show slightly better than with DNN. However, the SVR training time per sample is between 5.81 ms and 32.02 ms far longer than the DNN results. The smallest running time per sample is when C = 1 and ϵ = 0.2. This C result confirms that the smallest C will be computationally less expensive than the higher C [34].

**Figure 13:**
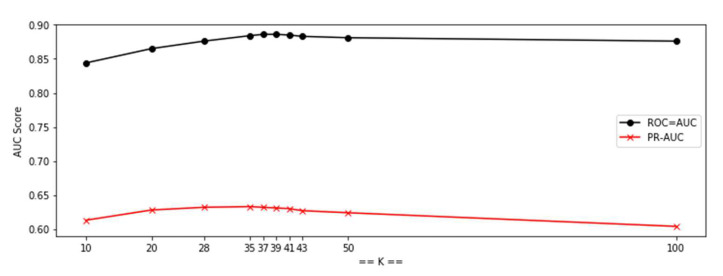
ROC and PR as a several results of k in KNN.

In the KNN experiments, the k is not swept from 2 to 50, as in [44], because this takes a lot of time. Here, manual selection, as described in Section 3.3, similar to binary search, was chosen to find the optimal k. The result is shown as in Figure 13. The ROC-AUC and PR-AUC are the upper and lower curves, respectively. From this graph the optimal k is found at k = 37. The k in KNN is selected based on the value of k, which produces the best ROC-AUC and PR-AUC from the testing results when run in Without-Clustering mode.

#### 4.2.2. Kmeans Experiment

Clustering at the beginning of the process can be used to group households that have similar profiles. Table 6 is obtained from the Kmeans experiments k = 2. Smart meters are divided into two clusters C0 and C1, with 77 and 123 smart meters IDs, respectively. There was a significant difference between C0 and C1. C0 shows better performance than C1, because it has more anomalous labels. The original C0 has 438 anomalous points, while C1 only has 159. Among all data in the cluster C0 and C1, the anomalous points are 1.6% and 0.4%, respectively.

In the case of DNN C0-128 as compared to D200-128 for all LA steps, the ROC-AUC and PR-AUC scores increase 10.2% and 48.4%, respectively. This increase increases true positive, decreases false positive, or false negative significantly. If number of smart meters are quite large, it should be possible for C1 members to be moved to the right cluster as long it has enough anomalous labels. To get another experiment sense, measurements of consumption patterns are proposed while using Dynamic Time Wrapping when compared to using Kmeans [14]. The benefit of using this clustering is mainly to reduce the clustering numbers because small shifts of electrical consumption should not be considered as a different cluster. In the next subsection, we show our proposed Kmeans*HDBSCAN experiment results that has the ability to increase the performance in the dominant cluster.

The results of clustering experiments trained with DNN, SVR, and KNN methods are shown in Table 6, Table 7 and Table 8. All three of these tables suffer from C1, so the results cannot be used in this clustering group. Of the three attempted methods, the average PR-AUC of C0-128 for DNN, SVR, and KNN is 0.837, 0.853, and 0.859. The results show a little difference with KNN is the best.

#### 4.2.3. Kmeans*HDBSCAN Experiment

In the clustering experiment, the Kmeans dataset was separated into two clusters C0 = 77 and C1 = 123 members. Kmeans assumes that the data are not contaminated with noise, so we need an algorithm that is able to detect the presence of noise data and group them separately. Density based algorithms, such as DBSCAN and HDBSCAN, can be utilized. In this experiment, HDBSCAN was chosen because it offers better performance than its predecessor.

**Figure 14:**
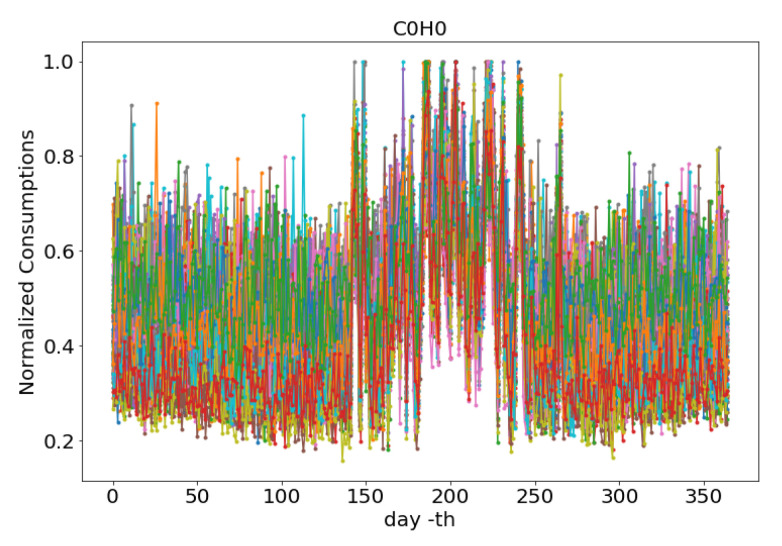
The Mix Pattern of C0H0.

The results of HDBSCAN clustering are in the form of noise groups and two clusters, H0 and H1. The sub total members of the HDBSCAN results are H0 = 80, H1 = 57 , and Noise = 63 members. The next process is by intersecting a combination of C0, and C1 with H0, and H1 to form four new sets as a result of this intersection. Table 9 shows the intersection members of C0H0, C0H1, C1H0, and C1H1 of 74, 0, 57, and 63, respectively. As seen in Figure 14, a uniform pattern is shown, when all members of the C0H0 are plotted.

**Figure 15:**
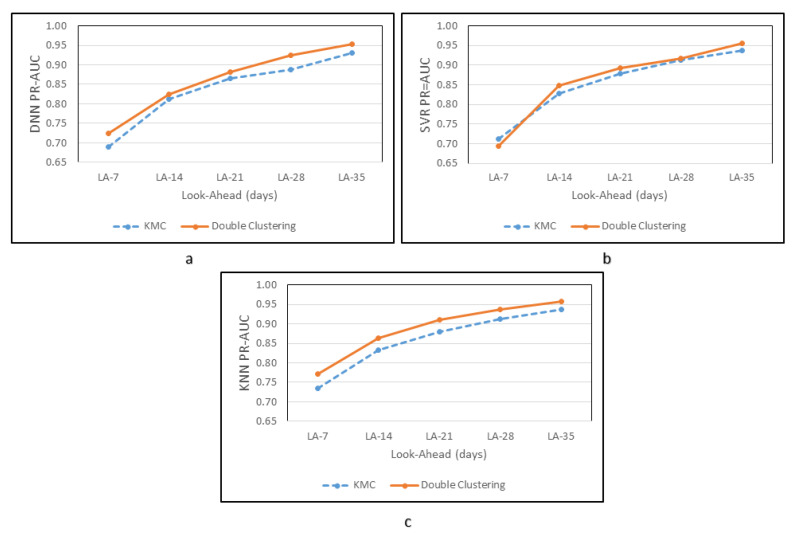
PR-AUC Comparison of Kmeans-C0 and Kmeans*HDBSCAN C0H0 for Machine Learning (**a**) DNN, (**b**) SVR, and (**c**) KNN.

Kmeans*HDBSCAN C0H0, as shown in Table 10, when compared to Kmeans-C0 at timesteps 128 for all LA, is able to increase the average performance of DNN, SVR, and KNN by 2.9%, 0.7%, and 3.3%, respectively. Previously the KNN had demonstrated an average of 0.859, but Kmeans*HDBSCAN caused an increase to 0.888. At the same time, DNN and SVR increased from 0.837 to 0.861, and 0.853 to 0.860, respectively. Figure 15 displays a comparison of DNN, SVR, and KNN, each with Kmeans and Kmeans*HDBSCAN types. Kmeans*HDBSCAN C0H0 shows better than Kmeans C0 with 128 timesteps.

**Figure 16:**
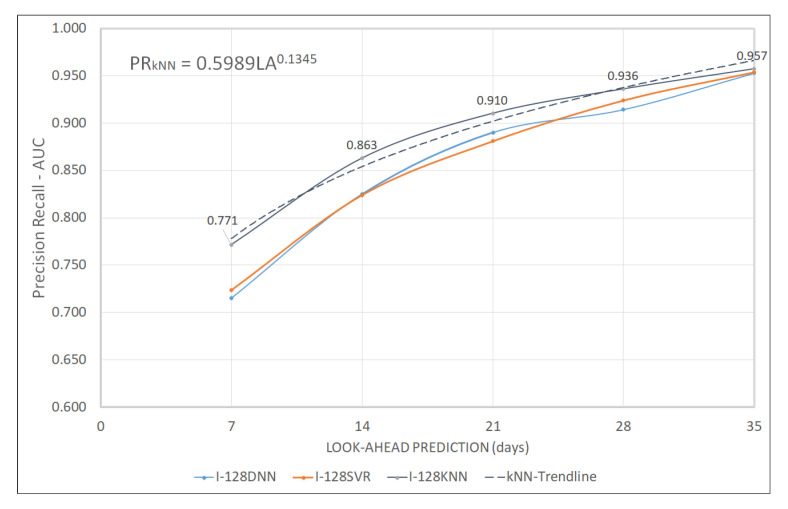
Kmeans*HDBSCAN Precision Recall Performance.

Figure 16 represents that, in this anomaly detection, KNN shows better than the similar performances of DNN and SVR.The results of KNN C-128 with LA-24 are generated from the power trendline equation as seen in Equation (1). The PR-AUC value for LA = 24 is 0.918 or the imperfect condition is 0.082. From the [14], the best 24-hour prediction was obtained from the smallest DTW error of 0.182. These results indicate that the proposed method is potentially better.

(1) $$PR_{kNN}=0.5989LA^{0.1345}$$

The SMOTE algorithm is also tested for timesteps = 128 and LA-14. The difference between the experimental steps and other algorithms is that SMOTE expands the minority sample to the same amount as the majority sample, so that the total number of samples is twice the majority sample. With a balanced sample, it is expected that the test sample will be in the area that has been developed. The test results are as seen in Table 10 C-128-SM. SMOTE is not able to increase the ROC and PR of the SVR, DNN, and KNN performance. The performance of SMOTE in this experiment is slightly lower than that of Kmeans*HDBSCAN. Another disadvantage of SMOTE is that the number of training samples increases. For DNN and SVR, the training time will be longer, while the inference time will be longer for the SVR and KNN algorithms. The SVR and KNN persistence files also increase to become 4.9 MB and 35.6 MB from the previous 3.5 MB and 23.8 MB, respectively.

**Figure 17:**
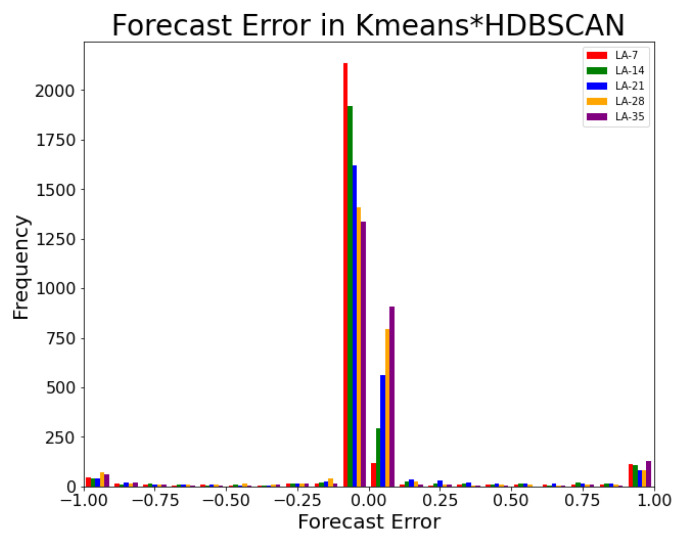
The Forecast Error Histogram of Kmeans*HDBSCAN in 20 bins.

The distribution of the forecast error from Kmeans*HDBSCAN with DNN, timesteps = 128, LA-14, and 2577 samples test data is shown in Figure 17. More than 90% of the prediction errors reside in the first left and right bins near the 0.00. Every sample is compared with label-0 or label-1, thus the minimum and maximum are –1.00 and 1.00. This information confirms the high score in True Positives and True Negatives.

#### 4.2.4. Performance Evaluation for All Clusters and Dominant Cluster

The performance of this system is tested and compared from the all of the clusters and based only on the dominant cluster. Testing the entire cluster is done by taking all of the values of true positive (TP), false positive (FP), true negative (TN), and false negative (FN) of each cluster model that is calculated from the experiment timesteps 128 and LA-14. In addition, the performance metrics of true positive rate (TPR), false positive rate (FPR), precision, recall, and F1 are calculated. In the same way for dominant clusters, but only one cluster is measured. Table 11 and Table 12 represent all of the performances of the machine learning applied here.

The F1-score performance of all clusters when compared to XGBOOST [45] as a baseline in the Without-Clustering type shows always higher. The application of Kmeans*HDBSCAN for all types of machines in this experiment always results in an F1 score that is slightly lower than the Kmeans, but is always higher than the Without-Clustering type. Recall or TPR from DNN, SVR, and KNN from Kmeans*HDBSCAN showed 0.018, 0.033, and 0.029 results better than Kmeans. This is due to a decrease in ΣFN or an increase of ΣTP.

In the dominant cluster, F1-score of Kmeans*HDBSCAN always shows 0.007, 0.016, and 0.010 higher than Kmeans for DNN, SVR, and KNN type of machines, respectively. The dominant cluster is a cluster that has more anomalies than other clusters. From 1052–1053 actual anomalies in the testing dataset, 749–769 data are actual anomalies in the dominant cluster. Table 12, shows that F1 scores for Kmeans*HDBSCAN C0H0 are always better than Kmeans C0 for all machine-learning in these experiments. This reflects that the combination of Kmeans and HDBSCAN is able to produce better performance than Kmeans alone in cluster with higher anomalies probability.

#### 4.2.5. Running on Edge Machine

As with techniques in machine learning, training normally requires an iteration process so that it takes longer than the inference process. A high number of parameters will affect the speed of inference. The model created by SVR is obtained after an iteration calculation process to look for w (weighted vector), which can separate the dataset using hyperplane. The number of parameters w is asymptotically as many as the amount of data X based on [33], which cites the work of [32] (i.e., w^T^.X). After the training, not all of the data points are important, but only those that become support vectors.

Comparison of the sizes of DNN, SVR, and kNN models states that the order from the smallest sizes is DNN, SVR, and KNN. KNN swallows all training data without processing beforehand, so that the training process is said to be the fastest, but it takes a little bit longer to load in the Raspberry Pi. DNN on the other hand, has a different technique, because it starts from a heuristic architectural design. Models are selected and determined from the beginning causing the size of the parameters to be predicted from the beginning and not depend on data size as in SVR or KNN.

**Figure 18:**
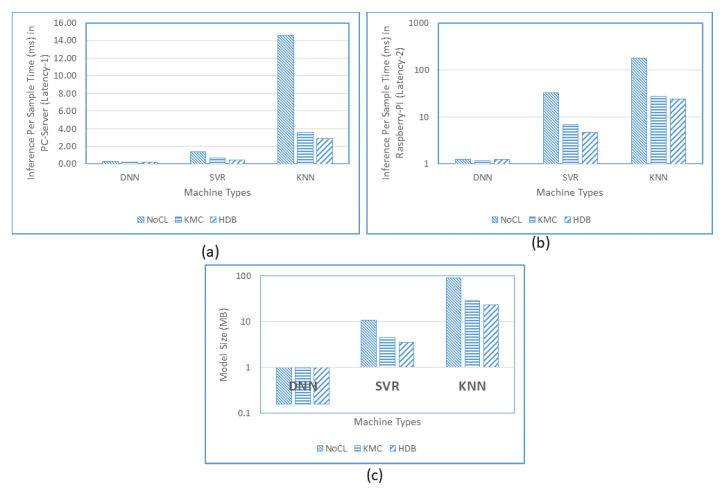
Inference Per Sample Time of Latency-1 (**a**), Latency-2 (**b**), and Model Size (**c**) Comparisons of the DNN, SVR, and KNN.

Table 13 and Figure 18 display the results of testing in tabular and graphical form. Testing is done on the server and edge to measure Latency-1 (Figure 18a) and Latency-2 (Figure 18b), respectively, and measure the size of the file persistent (Figure 18c) as the implementation of machine learning. Testing DNN in the PC-server is faster than SVR and KNN because DNN is supported by NVIDIA-GPU, while SVR and KNN that are built on the sklearn [34] library only optimized in the host processor. Sklearn does not yet provide a version that can access the GPU when run-time on PC and Servers. Latency-1 of DNN, SVR, and KNN in Kmeans*HDBSCAN if the test runs on PC-server shows 0.23 ms, 0.47 ms, and 2.90 ms. In the edge for Kmeans*HDBSCAN, DNN displays results with inference speed performance 3.8 and 19.6 times faster than SVR and KNN, respectively. In other words, the Latency-2 of DNN, SVR, and KNN in the Raspberry Pi are, respectively, 1.24 ms 4.76 ms, and 24.34 ms. DNN is also always faster for the case of clustering with Kmeans compared to SVR and KNN. But the inference speed of SVR and KNN in clustering mode is increasing very rapidly. This is consistent with the theory that both the SVR and KNN models depend on the amount of training data. While in DNN the inference time is relatively fixed, because the model does not depend on the amount of data trained. Clustering techniques reduce the amount of training data, so that the calculation of support vectors being sought and searching for data samples around the sample point reduces. DNN is still far faster than SVR and KNN despite this time reduction for SVR and KNN.

Table 13 and Figure 18c also show the model file size with the DNN model as the smallest, followed by SVR and KNN. The DNN model is stored in .h5 and .json files, while the SVR and KNN use .joblib. DNN in Without-Clustering mode is 69 and 618 times smaller than SVR and KNN, respectively, because it is a pre-designed model with several tests with parameters and options as variables. However, after clustering, the persistence file size is still much larger than the DNN. The small persistence file speeds up the process of changing the model sent by the cloud when the old model is not suitable and needs to be replaced. DNN is far better than SVR or KNN in the case of latency and model file size.

For each inference process in Raspberry Pi is around 1.25 ms with a communication delay of about 10ms that allows the processing of 89 smart meter data/second. For a larger area of sensor network with a time step of 30 min and a transmission duty cycle of 1/100, effectively transmission can be done in 30 × 60 s × 1/100 or 18 s. During this, 18 s and with 89 inferences/second, totally this simple edge can process 1600 smart meters that can be handled by the edge at a time. If five retransmission is allowed then maximum 1600/5 or 320 smart meters can be served per 30 min. In our dataset, there are 200 smart meters identifiers; therefore, this Raspberry Pi is enough to satisfy this specification. Thus, even though the latency in Edge is five times higher than in the PC-server, this specification still can be fulfilled.

In Table 13, DNN and KNN show more linear results than SVR. SVR Without Clustering processing on a PC took 1.38 ms, while, on Raspberry PI, it was 33.11 ms. The possible answers is because the SVR library (Sklearn) optimizes the solution based on the underlying hardware architecture. The i7 architecture is more complex than Arm-based architecture, which can possibly make the processing much faster. In addition, the complexity of the SVR O(n${}_{features}$× n${}^{2}$${}_{samples}$) is largely determined by the number of samples. Accordingly, the latency obtained cannot be linear as a result of the number of samples and architectural models.

## 5. Conclusions

There are four conclusions that can be drawn from all of the experiments that have been carried out. First, we have proposed an Edge deployment model to overcome the excessive latency of the centralized model. Edge gets data sent from the smart meter with a client-server model via Ethernet as a simulation of data transmission. In the inference time/sample and persistant file size tests, DNN outperformed the SVR and KNN for all types of Without-Clustering, Kmeans, and Kmeans*HDBSCAN experiments. Second, the experimental results of the PR-AUC performance of the anomaly detection for the Without-Clustering, Kmeans, and Kmeans*HDBSCAN types showed that KNN was always slightly better and was followed by SVR and DNN. Third, the multitiered solution that has been applied in each experiment shows the tendency to be intuitively correct, namely, the timesteps of the extended sliding window and the widened look-ahead label will always improve the performance of PR-AUC. Finally, a comparison test on the dominant clusters of Kmeans*HDBSCAN and Kmeans shows that the dominant clusters of Kmeans*HDBSCAN are always slightly better to those of Kmeans alone.

## Figures and Tables

**Table 1:** General Comparison of Deep Neural Networks (DNN), Support Vector Regression (SVR), and k-Nearest Neighbors (KNN) Algorithms.

Characteristic	DNN	SVR	KNN
Model Determination	Desainer layout (i.e., model.summary [30])	Linear Function, Kernel trick, and Optimization Problem [31]	k value
Training Goal	Update node weights	Find the support vectors [32,33]	Remember all training data [34]
Persistence Size	Fixed predetermined model	Size of support vectors	Fixed based on training data
Training Time	Slow	Medium	Fast
Inference Time	Fast	Medium	Slow

**Table 2:** Hardware and Software for Training and Testing Environment.

Name	PC-Server	Raspberry Pi 3B+
CPU	Intel (R) i7-7700	Arm Cortex-A53
GPU	NVIDIA GTX 1060	-
RAM	16 GB	1 GB
Operating System	Windows 10	Raspbian Stretch
Python	3.6.6	3.5.3
Tensorflow	1.10.0	1.8
Keras	2.2.2	2.2.4
Scikit-learn	0.22.2	0.22.2

**Table 3:** ROC and PR as test results of one layer LSTM with various parameters changes.

Experiments	ROC-AUC			PR-AUC			TOTAL-AUC		
**LSTM-1L**	**Tanh**	**Sigmoid**	**ReLU**	**Tanh**	**Sigmoid**	**ReLU**	**Tanh**	**Sigmoid**	**ReLU**
N32-Cv16	0.829	0.821	0.836	0.498	0.485	0.517	1.327	1.306	1.353
N32-Cv32	0.838	0.845	0.866	0.527	0.532	0.563	1.365	1.378	1.429
N32-Cv64	0.852	0.882	0.859	0.548	0.583	0.563	1.400	**1.465**	1.423
N64-Cv16	0.844	0.804	0.839	0.532	0.462	0.489	1.376	1.266	1.328
N64-Cv32	0.873	0.814	0.852	0.575	0.481	0.541	1.448	1.295	1.393
N64-Cv64	0.847	**0.885**	0.858	0.541	0.554	**0.595**	1.387	1.439	1.453
N256-Cv16	0.836	0.852	0.855	0.504	0.485	0.551	1.339	1.337	1.405
N256-Cv32	0.838	0.846	0.844	0.550	0.523	0.545	1.388	1.368	1.389
N256-Cv64	0.824	0.842	0.866	0.538	0.466	0.550	1.362	1.308	1.417

N32-Cv64 = 32 Unit LSTM, Convolution 1D layer (4, 8, 16, 32, 64).

**Table 4:** ROC and PR as the test results of one layer LSTM with various timesteps.

Experiments	ROC-AUC					PR-AUC				
**1L32N64**	**LA-7**	**LA-14**	**LA-21**	**LA-28**	**LA-35**	**LA-7**	**LA-14**	**LA-21**	**LA-28**	**LA-35**
D200-032	0.780	0.780	0.783	0.779	0.783	0.394	0.475	0.522	0.549	0.577
D200-064	0.829	0.819	0.823	0.821	0.820	0.456	0.529	0.567	0.591	0.616
**D200-128**	0.860	0.856	0.853	0.846	0.837	0.472	**0.549**	0.575	0.596	0.629

LA = Look-Ahead, D200-128 = 200 Smart Meters ID, Single LSTM layer with 128 timesteps.

**Table 5:** ROC and PR of SVR with various parameter C and ϵ.

Experiments	ROC-AUC				PR-AUC			
**C, ϵ**	**0.1**	**0.2**	**0.3**	**0.4**	**0.1**	**0.2**	**0.3**	**0.4**
**1**	0.850	0.855	0.859	0.864	0.582	**0.588**	0.600	0.612
2	0.854	0.859	0.864	0.871	0.602	0.606	0.612	0.617
3	0.854	0.862	0.868	0.874	0.608	0.611	0.616	0.619
4	0.853	0.865	0.871	0.876	0.610	0.615	0.617	0.618

C, ϵ = SVR parameter.

**Table 6:** ROC-AUC and PR-AUC as test results of Two Clusters Kmeans in DNN with various timesteps.

Experiments	ROC-AUC					PR-AUC				
**Tanh-Sig**	**LA-7**	**LA-14**	**LA-21**	**LA-28**	**LA-35**	**LA-7**	**LA-14**	**LA-21**	**LA-28**	**LA-35**
C0-032	0.889	0.878	0.900	0.870	0.899	0.589	**0.710**	0.782	0.775	0.842
C0-064	0.929	0.929	0.924	0.931	0.930	0.700	**0.794**	0.831	0.878	0.895
**C0-128**	0.922	0.936	0.939	0.938	0.953	**0.689**	**0.813**	**0.865**	**0.887**	**0.931**
C1-032	0.667	0.669	0.665	0.683	0.709	0.049	0.076	0.101	0.142	0.181
C1-064	0.705	0.723	0.738	0.727	0.745	0.062	0.132	0.165	0.183	0.255
C1-128	0.739	0.722	0.744	0.759	0.751	0.080	0.115	0.171	0.227	0.261

**Table 7:** ROC and PR C = 1 and ϵ = 0.2 as test results of Two Clusters Kmeans in SVR with various timesteps.

Experiments	ROC-AUC					PR-AUC				
**C = 1, ϵ = 0.2**	**LA-7**	**LA-14**	**LA-21**	**LA-28**	**LA-35**	**LA-7**	**LA-14**	**LA-21**	**LA-28**	**LA-35**
C0-032	0.901	0.914	0.919	0.920	0.923	0.674	0.782	0.829	0.854	0.879
C0-064	0.899	0.921	0.929	0.932	0.934	0.695	0.808	0.857	0.880	0.900
**C0-128**	0.915	0.930	0.938	0.948	0.956	**0.711**	**0.827**	**0.879**	**0.912**	**0.937**
C1-032	0.667	0.684	0.697	0.703	0.703	0.052	0.099	0.135	0.174	0.203
C1-064	0.713	0.738	0.743	0.745	0.751	0.075	0.152	0.206	0.254	0.301
C1-128	0.748	0.764	0.774	0.782	0.796	0.113	0.181	0.235	0.301	0.351

**Table 8:** ROC and PR K = 37 as test results of Two Clusters Kmeans in KNN with various timesteps.

Experiments	ROC-AUC					PR-AUC				
**K = 37**	**LA-7**	**LA-14**	**LA-21**	**LA-28**	**LA-35**	**LA-7**	**LA-14**	**LA-21**	**LA-28**	**LA-35**
C0-032	0.933	0.928	0.925	0.922	0.925	0.693	0.784	0.829	0.854	0.885
C0-064	0.935	0.930	0.931	0.933	0.937	0.725	0.814	0.858	0.883	0.907
**C0-128**	0.940	0.942	0.947	0.952	0.957	**0.734**	**0.833**	**0.880**	**0.912**	**0.937**
C1-032	0.667	0.690	0.698	0.704	0.711	0.039	0.084	0.123	0.150	0.180
C1-064	0.676	0.719	0.725	0.730	0.738	0.063	0.118	0.147	0.177	0.214
C1-128	0.696	0.730	0.757	0.772	0.788	0.084	0.150	0.203	0.260	0.321

LA = Look-Ahead, C0-032: C0 = Cluster-0, Sliding Windows with 32 timesteps.

**Table 9:** Kmeans*HDBSCAN Candidate Cluster Determination.

Kmeans	HDBSCAN	Kmeans ∩ HDBSCAN
C0 = 77	H0 = 80	74
C0 = 77	H1 = 57	0
C1 = 123	H0 = 80	6
C1 = 123	H1 = 57	57
	Noise = 63	63

**Table 10:** ROC and PR of DNN, SVR, and KNN after Kmeans*HDBSCAN C0H0 in various timesteps.

Experiments	ROC-AUC					PR-AUC				
	**LA-7**	**LA-14**	**LA-21**	**LA-28**	**LA-35**	**LA-7**	**LA-14**	**LA-21**	**LA-28**	**LA-35**
DNN										
C-032	0.906	0.925	0.925	0.923	0.921	0.655	0.747	0.807	0.845	0.876
C-064	0.924	0.934	0.938	0.941	0.943	0.716	0.812	0.861	0.895	0.922
**C-128**	0.913	0.934	0.945	0.957	0.967	**0.724**	**0.824**	**0.881**	**0.924**	**0.954**
C-128-SM	-	0.932	-	-	-	-	**0.816**	-	-	-
SVR										
C-032	0.911	0.916	0.907	0.913	0.910	0.642	0.764	0.807	0.847	0.869
C-064	0.906	0.937	0.938	0.931	0.938	0.668	0.822	0.877	0.885	0.900
**C-128**	0.927	0.945	0.950	0.954	0.966	**0.694**	**0.847**	**0.893**	**0.917**	**0.955**
C-128-SM	-	0.943	-	-	-	-	**0.845**	-	-	-
KNN										
C-032	0.944	0.939	0.936	0.934	0.935	0.727	0.803	0.851	0.878	0.907
C-064	0.945	0.942	0.942	0.944	0.945	0.745	0.828	0.872	0.903	0.924
**C-128**	0.947	0.952	0.958	0.963	0.968	**0.771**	**0.863**	**0.910**	**0.936**	**0.957**
C-128-SM	-	0.946	-	-	-	-	**0.852**	-	-	-

LA = Look-Ahead, C-032: C = Sliding Windows with 32 timesteps, SM = SMOTE.

**Table 11:** All Clusters Performance Evaluation of Various Machines and Types for Timesteps-128 and LA-14.

Machine	Type	ΣTP	ΣFP	ΣTN	ΣFN	TPR	FPR	Precision	Recall	F1
XGBOOST	Without-Clustering	555	447	7460	498	0.527	0.057	0.554	0.527	0.540
DNN	Without-Clustering	616	521	7385	436	0.586	0.066	0.542	0.586	0.563
DNN	Kmeans	616	292	7614	437	0.585	0.037	0.699	**0.585**	0.637
DNN	Kmeans*HDBSCAN	635	360	7545	417	0.603	0.046	0.638	**0.603**	0.620
SVR	Without-Clustering	637	250	7655	416	0.605	0.032	0.718	0.605	0.657
SVR	Kmeans	617	211	7695	436	**0.586**	0.027	0.758	**0.586**	0.661
SVR	Kmeans*HDBSCAN	650	266	7638	400	0.619	0.034	0.710	**0.619**	0.661
KNN	Without-Clustering	629	270	7635	424	0.597	0.034	0.700	0.597	0.644
KNN	Kmeans	615	198	7709	438	0.584	0.025	0.765	**0.584**	0.662
KNN	Kmeans*HDBSCAN	645	272	7632	407	0.613	0.034	0.704	**0.613**	0.655

ΣTP = Total True Positive, ΣFP = Total False Positive, ΣTN = Total True Negative, ΣFN = Total False Negative, TPR = True Positive Rate = ΣTP/(ΣTP + ΣFN), FPR = False Positive Rate = ΣFP/(ΣFP + ΣTN), Precision = ΣTP/(ΣTP + ΣFP), Recall = ΣTP/(ΣTP + ΣFN), F1 = 2 × PrecisionxRecall/(Precision + Recall) LA = Look-Ahead.

**Table 12:** Dominant Cluster Performance Evaluation of Various Machines and Types for Timesteps-128 and LA-14.

	Dominant									
Machine	Cluster-Only	TP	FP	TN	FN	TPR	FPR	Precision	Recall	F1
DNN	C0	616	291	2389	153	0.801	0.109	0.682	0.801	**0.737**
DNN	C0H0	596	265	2300	153	0.796	0.103	0.699	0.796	**0.744**
SVR	C0	617	211	2469	153	0.802	0.079	0.746	0.802	**0.773**
SVR	C0H0	616	196	2369	133	0.822	0.076	0.758	0.822	**0.789**
KNN	C0	614	197	2484	155	0.799	0.073	0.761	0.799	**0.780**
KNN	C0H0	611	191	2374	138	0.816	0.074	0.765	0.816	**0.790**

TP = True Positive, FP = False Positive, TN = True Negative, FN = False Negative, TPR = True Positive Rate = TP/(TP + FN), FPR = False Positive Rate = FP/(FP + TN), Precision = TP/(TP + FP), Recall = TP/(TP + FN), F1 = 2 × PrecisionxRecall/(Precision + Recall), LA = Look – Ahead. C0= Dominant Cluster of Kmeans, C0H0 = Dominant Cluster of Kmeans*HDBSCAN

**Table 13:** Testing Time, Frequency, and Model Size Comparisons of the DNN, SVR, and KNN.

Machine	Inference/Sample (ms)			Inference Frequency (/s)			Size of the Model (KB)		
**A. Server+GPU (Latency-1)**	**DNN**	**SVR**	**KNN**	**DNN**	**SVR**	**KNN**	**DNN**	**SVR**	**KNN**
1. Without-Clustering	**0.25**	1.38	14.56	4,000	725	69	**159**	**10,807**	**92,501**
2. Kmeans	**0.22**	0.65	3.59	4,525	1,550	278	159	4,517	28,849
3. Kmeans*HDBSCAN	**0.23**	**0.47**	**2.90**	4,425	2,123	345	159	3,524	23,794
**B. Raspberry Pi (Latency-2)**									
1. Without-Clustering	1.26	33.11	176.17	794	30	6	159	10,807	92,501
2. Kmeans	1.18	6.05	27.87	847	165	36	159	4,517	28,849
3. Kmeans*HDBSCAN	**1.24**	**4.76**	**24.34**	810	210	41	159	3,524	23,794

Without-Clustering = D200, Kmeans = C0, Kmeans*HDBSCAN = C0H0.

## Abbreviations

The following abbreviations are used in this manuscript:

AMI	Advanced Metering Infrastructure
MDMS	Meter Data Management Systems
LF	Load Forecasting
LSTM	Long Short Term Memory
DNN	Deep Neural Network
KNN	k-Nearest Neighbors
SVR	Support Vector Regression
SVM	Support Vector Machine
Conv1D	Convolution one Dimension
SMOTE	Synthetic Minority Oversampling Technique
ADASYN	Adaptive Synthetic sampling
Kmeans	k-means clustering
HDBSCAN	Hierarchical Density-Based Spatial Clustering of Applications with Noise
SC	Silhouette Coefficient
ROC	Receiver Operating Characteristics
AUC	Area Under the Curve
PR	Precision Recall
TPR	True Positive Rate
FPR	False Positive Rate
ACC	Accuracy
TP	True Positive
TN	True Negative
FP	False Positive
FN	False Negative
